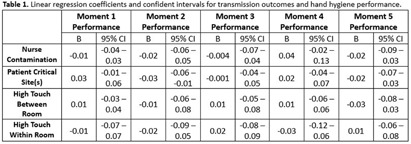# Connecting Pathogen Transmission and Performance of the WHO’s ‘My 5 Moments of Hand Hygiene’ in a High-Fidelity Simulation

**DOI:** 10.1017/ash.2024.244

**Published:** 2024-09-16

**Authors:** Paige Gannon, Rebecca MacKay, Kari Love, Bradley Weaver, Kylie Burke, Victoria Dotto, Joselyn Garcia, Angelina Luciano, Brandon Berryhill, Joel Mumma

**Affiliations:** Healthcare Human Factors Lab, Emory University; Emory University; Emory Healthcare; Healthcare Human Factors Lab; Chenega Enterprise Systems and Solutions; 1987

## Abstract

**Background:** The World Health Organization launched ‘Your 5 moments for hand hygiene’ to identify when healthcare workers should perform hand hygiene to reduce healthcare-associated infections (HAIs). Performing hand hygiene correctly is necessary to decrease pathogen transfer, though little research has assessed the effectiveness of all 5 moments. **Methods:** Registered nurses (n=42) participated in a standardized, one-hour high-fidelity patient care simulation that were recorded via a head-mounted camera. The simulation involved two patients, each requiring four clinical care tasks (e.g., indwelling Foley catheter insertion, stool sample collection). Transmission data was obtained from the simulations using four genetic variants of bacteriophage λ. Before each simulation, variants were applied to unique locations on two manikins: patient A’s wound, patient A’s stool, patient B’s groin, and patient B’s stool. After each simulation, we sampled the patients, nurse, and high-touch environmental surfaces to determined bacteriophage identity of positive samples. For each moment, hand hygiene performance was the total time the nurse practiced hand hygiene across opportunities over the total recommended time (15 seconds per opportunity). Positive samples were categorized as 1) nurse contamination, 2) patient critical site(s) contamination, 3) high touch surface contamination from the same patient, or 4) high touch surface contamination from the other patient. To compare nurse’s performance of each of the 5 moments, we used a Friedman test and then a Wilcoxon test for pairwise comparisons. To assess the relationship between the four types of transmission outcomes and hand hygiene performance of the 5 moments, we performed linear regressions and calculated 95% confidence intervals by bootstrapping the original cases. **Results:** Performance of moments 1 (Before patient contact: 9.49%), 4 (After patient contact: 13.11%), and 5 (After contact with patient’s surroundings: 13.66%) were significantly higher than moments 2 (Before clean or aseptic task: 2.72%) and 3 (After bodily fluid exposure: 4.22%; p < 0 .05). Moment 2 perfomance, furthermore, was significantly lower than moment 3 (Figure 1). Only moment 2’s performance was significantly related to transmission; specifically, performance was negatively related to critical site contamination (B= -0.03, CI 95%: -0.06 – -0.01); Table 1. **Conclusions:** Moment 2performance was the lowest of all 5 moments and was the only moment that demonstrated evidence of relationship with pathogen transmission, specifically critical site contamination. Of all the 5 moments, this moment is most directly related to HAIs. Further research should investigate why moment 2 performance is so low.

**Figure:**
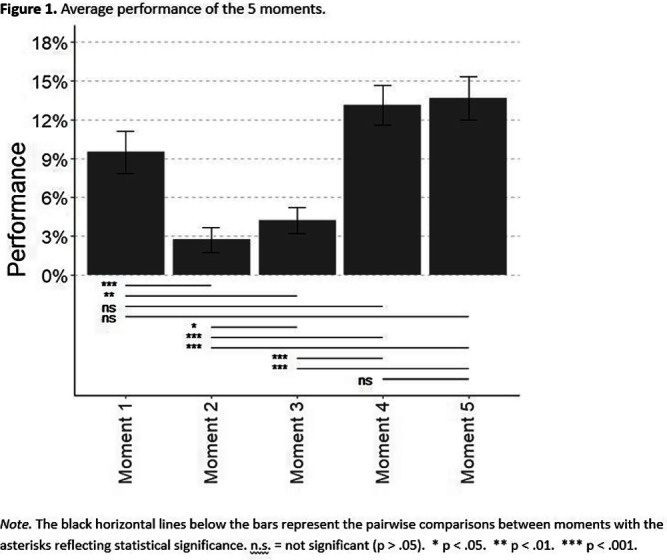

**Figure:**